# Reply to the letter “Understanding lactate and its clearance during extracorporeal membrane oxygenation for supporting refractory cardiogenic shock patients”

**DOI:** 10.1186/s12872-023-03273-0

**Published:** 2023-05-12

**Authors:** Fernando Luís Scolari, Daniel Schneider, Débora Vacaro Fogazzi, Miguel Gus, Marciane Maria Rover, Marcely Gimenes Bonatto, Gustavo Neves de Araújo, André Zimerman, Daniel Sganzerla, Lívia Adams Goldraich, Cassiano Teixeira, Gilberto Friedman, Carisi Anne Polanczyk, Luis Eduardo Rohde, Regis Goulart Rosa, Rodrigo Vugman Wainstein

**Affiliations:** 1 Research Projects Office, Division of Cardiology, Hospital Moinhos de Vento (HMV), Hospital de Clínicas de Porto Alegre (HCPA), Rua Ramiro Barcelos 630, Porto Alegre, RS 90035-001 Brazil; 2 Research Projects Office, HMV, Rua Ramiro Barcelos 630, Porto Alegre, RS 90035-001 Brazil; 3 Division of Cardiology, HMV, Rua Tiradentes, 333, Porto Alegre, RS 90560-030 Brazil; 4 Division of Cardiology, Heart Failure and Transplant Division, HMV, Instituto de Cardiologia – Fundação Universitária de Cardiologia, Av. Princesa Isabel, 395, Porto Alegre, RS 90040- 371 Brazil; 5 Cardiology Department, Transplant Division, Irmandade Hospital da Santa Casa de Misericórdia de Curitiba, Praça Rui Barbosa, 694, Curitiba, PR 80010-030 Brazil; 6 Federal University of Rio Grande do Sul, Rua Ramiro Barcelos, Porto Alegre, RS 2350, 90035-007 Brazil; 7 Division of Cardiology, HCPA, Rua Ramiro Barcelos, Porto Alegre, Porto Alegre, 2350, 90035-007 RS, RS Brazil; 8 Division of Critical Care Medicine, HMV, R. Tiradentes, 333, Porto Alegre, 90560-030 Brazil; 9 Division of Critical Care Medicine, HCPA, Rua Ramiro Barcelos 630, Porto Alegre, 90035-001 Brazil; 10 Division of Cardiology, HMV, HCPA, UFRGS, Associate Professor of Medicine, Postgraduate Program in Health Sciences: Cardiology and Cardiovascular Sciences, UFRGS, R. Tiradentes, 333, Porto Alegre, 90560-030 Brazil; 11 Research Projects Office, Division of Cardiology, HMV, HCPA, Postgraduate Program in Health Sciences: Cardiology and Cardiovascular Sciences, UFRGS, Rua Ramiro Barcelos 630, 10º andar, Porto Alegre, RS 90035-004 Brazil

## Abstract

This is a reply to the letter titled “Understanding lactate and its clearance during extracorporeal membrane oxygenation for supporting refractory cardiogenic shock patients” by Eva Rully Kurniawati et al. In response to the concerns raised about our paper published in BMC Cardiovascular Disorders, titled “Association between serum lactate levels and mortality in patients with cardiogenic shock receiving mechanical circulatory support: a multicenter retrospective cohort study,“ we have addressed the confounding bias on the population included and the use of VA-ECMO and Impella CP. Furthermore, we have provided new data on the correlation of oxygen supply and lactate levels at admission of cardiogenic shock.

## Reply

The systemic hypoperfusion associated with cardiogenic shock (CS) triggers several inflammatory pathways resulting in systemic multi-organ failure and death [1]. Mortality rates range from 28 to 50% depending on the etiology and population included, but the overall mortality rate has not changed in the past decade [2]. Mechanical circulatory support (MCS) has been used as a strategy to restore tissue perfusion allowing myocardial recovery, or as a bridge to long-term left ventricular assist devices or orthotopic heart transplant [1, 2]. Despite the suggested benefit on observational studies and mortality reduction with current strategies, no randomized clinical trial has shown a survival benefit with MCS. Our study included patients supported with extracorporeal membrane oxygenation (ECMO) and/or Impella CP to support CS patients with various etiologies [2]. This multicentric cohort focused on lactate kinetics, with lactate levels were evaluated at the time of support initiation and after 1 h, 6 h, 12 and 24 h [3]. As expected, lactate levels were associated with survival, whereas lactate clearance 24 h showed the strongest association.

We appreciate the interest of Kurniawati ER and colleagues in our study and thank them for their insightful comments. CS leads to multi-organ failure due to an imbalance in tissue oxygen delivery resulting in increased lactate levels [4]. It is worth noting that our CS patients did not have any associated conditions, such as diabetes ketoacidosis or smoke inhalation, which could confound the interpretation of lactate kinetics. The mechanical principles of ECMO and Impella CP catheter differ [1]. While Impella CP is an axial pump that unloads the left ventricle by pulling blood from the chamber into the ascending aorta, ECMO in the venoarterial (VA) configuration drains blood from the right atrium, passes it through an oxygenation membrane, and delivers it to the aorta. Therefore, ECMO may be the MCS of choice when hypoxia is a concern at the time of CS presentation due to its respiratory support. At the time of MCS implant, there were no significant differences in pO2 between VA-ECMO and Impella CP [88.9 (70.8–135.0) mmHg vs. 113.0 (71.0-130.0) mmHg, p = 0.348] in our cohort. Similarly, lactate levels at presentation were comparable between VA-ECMO and Impella CP patients [5.1 (2.5–9.5) vs. 6.4 (3.5–9.9), p = 0.632]. Survivors and non-survivors had similar pO2 levels at presentation [121 (72.9-133.7) vs. 88.9 (66.7-132.2), p = 0.232] and 24 h after MCS initiation [98.8 (82.1-141.5) vs. 124.5 (86.8-148.7), p = 0.353].

**Fig. 1:**
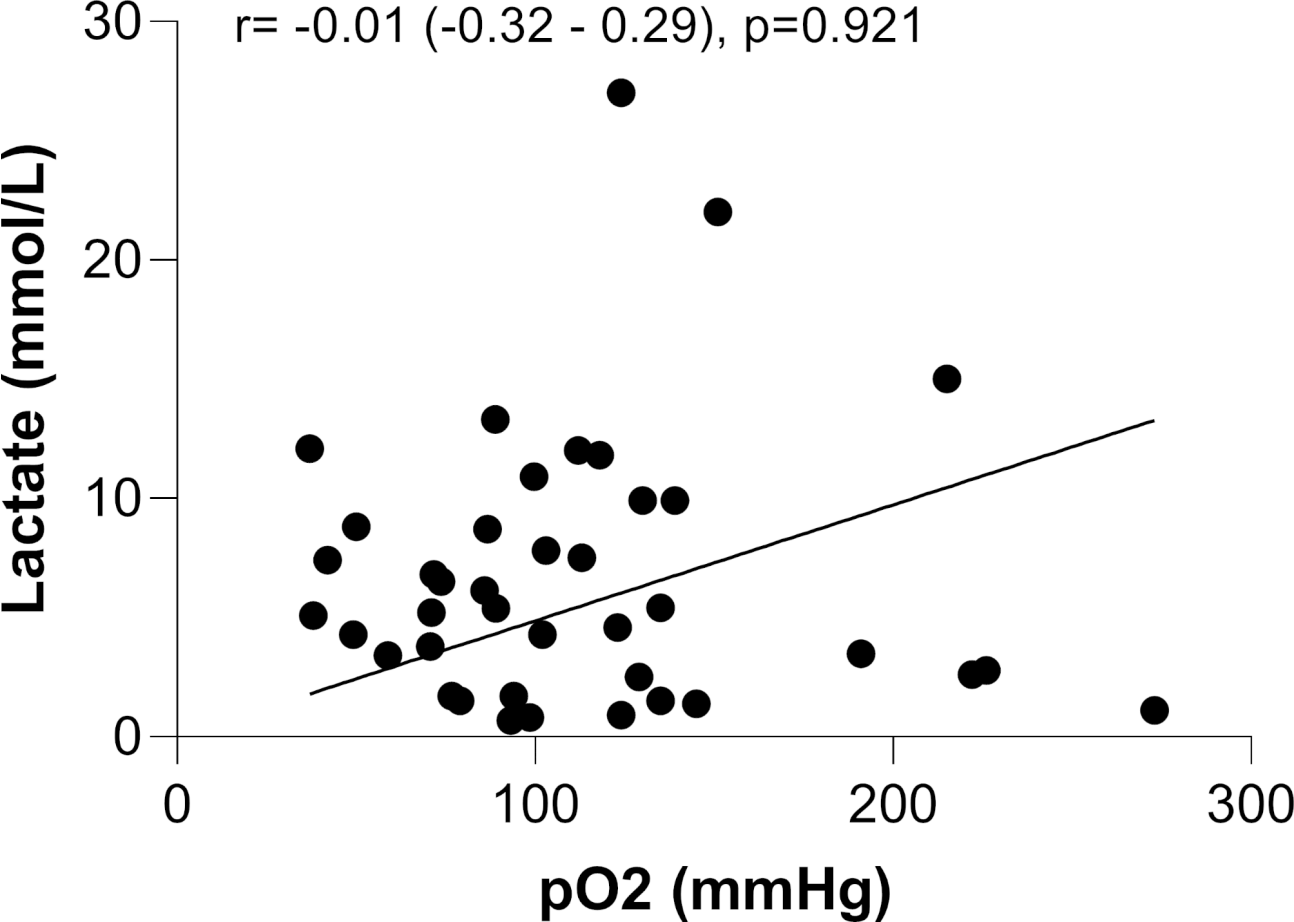
Linear correlation of the lactate level with pO2 at the admission of cardiogenic shock patients

In their comment, Kurniawati ER and colleagues suggested that higher initial lactate levels in patients with CS may require a higher oxygen delivery to repay the oxygen debt. While this may be true in terms of oxygen supply at the tissue level, it is important to note that cardiogenic shock reduces tissue oxygen supply due to hypoperfusion, despite optimal oxygenation [4]. To investigate this further, we performed a Pearson correlation analysis between the pO2 levels and lactate levels at the time of presentation (Fig. 1). Our results revealed no significant correlation [r= -0.01 (-0.32-0.29) p = 0.921].

Understanding lactate kinetics during MCS is complex. Despite the fact that ECMO can provide higher respiratory support, our study did not find any significant differences in the arterial partial pressure of oxygen (pO2) levels at presentation or after 24 h between VA-ECMO and Impella CP-supported patients. However, it is important to consider the limitations of these two MCS devices in terms of the amount of support they can provide. While Impella CP can provide up to 4.3 L per minute, VA-ECMO can provide up to 3–7 L per minute. It is worth noting that in our project, Impella CP was recommended for patients without hypoxia and with less severe CS, which reduces the potential bias of lower support for these patients.

Moreover, the inflammatory cascade triggered by CS may also interfere with lactate kinetics and oxygen tissue demands. However, the inflammatory response in CS is still a matter of ongoing discussion. Recent studies have highlighted the role of clonal hematopoiesis in dysregulating inflammatory cytokines in CS, leading to poorer outcomes [5]. Therefore, it is imperative to further investigate these mechanisms to better understand the pathophysiology of CS and to improve outcomes for affected patients.

The complex interplay between lactate kinetics in CS and its interaction with MCS remains a subject of ongoing debate. Lactate kinetics play an important role in the pathophysiology of CS, and the use of MCS devices can improve lactate kinetics in these patients. Further research is needed to fully understand the mechanisms underlying these effects and to optimize the use of MCS in the treatment of CS.

## Data Availability

Not applicable.
